# Prognostic value of inflammatory nutritional scores in locally advanced esophageal squamous cell carcinoma patients undergoing neoadjuvant chemoimmunotherapy: a multicenter study in China

**DOI:** 10.3389/fonc.2024.1279733

**Published:** 2024-02-15

**Authors:** Jinxin Xu, Zhinuan Hong, Yingjie Cai, Zhen Chen, Jingping Lin, Xi Yuan, Shuchen Chen, Jinbiao Xie, Mingqiang Kang, Sunkui Ke

**Affiliations:** ^1^ Department of Thoracic Surgery, Zhongshan Hospital Xiamen University, Xiamen, China; ^2^ Fujian Medical University, Fuzhou, China; ^3^ Department of Thoracic Surgery, Fujian Medical University Union Hospital, Fuzhou, China; ^4^ Key Laboratory of Cardio-Thoracic Surgery(Fujian Medical University), Fujian Province University, Fuzhou, China; ^5^ Key Laboratory of Ministry of Education for Gastrointestinal Cancer, Fujian Medical University, Fuzhou, China; ^6^ Fujian Key Laboratory of Tumor Microbiology, Fujian Medical University, Fuzhou, China; ^7^ Department of Cardiothoracic Surgery, The Affiliated Hospital of Putian University, Putian, China; ^8^ Department of Cardiothoracic Surgery, Putian Pulmonary Hospital, Putian, China; ^9^ The First Affiliated Hospital of Xiamen University, Xiamen, China; ^10^ Fujian Rongcheng Judicial Compulsory Isolation Drug Rehabilitation Center, Fuzhou, China

**Keywords:** esophageal squamous cell carcinoma, neoadjuvant chemoimmunotherapy, inflammatory nutritional scores, hemoglobin, albumin, lymphocyte, and platelet, major pathological response, disease-free survival

## Abstract

**Objective:**

This study investigates the prognostic significance of inflammatory nutritional scores in patients with locally advanced esophageal squamous cell carcinoma (LA-ESCC) undergoing neoadjuvant chemoimmunotherapy.

**Methods:**

A total of 190 LA-ESCC patients were recruited from three medical centers across China. Pre-treatment laboratory tests were utilized to calculate inflammatory nutritional scores. LASSO regression and multivariate logistic regression analyses were conducted to pinpoint predictors of pathological response. Kaplan-Meier and Cox regression analyses were employed to assess disease-free survival (DFS) prognostic factors.

**Results:**

The cohort comprised 154 males (81.05%) and 36 females (18.95%), with a median age of 61.4 years. Pathological complete response (pCR) was achieved in 17.38% of patients, while 44.78% attained major pathological response (MPR). LASSO and multivariate logistic regression analyses identified that hemoglobin, albumin, lymphocyte, and platelet (HALP) (P=0.02) as an independent predictors of MPR in LA-ESCC patients receiving neoadjuvant chemoimmunotherapy. Kaplan-Meier and log-rank tests indicated that patients with low HALP, MPR, ypT1-2, ypN0 and, ypTNM I stages had prolonged DFS (P < 0.05). Furthermore, univariate and multivariate Cox regression analyses underscored HALP (P = 0.019) and ypT (P = 0.029) as independent predictive factors for DFS in ESCC.

**Conclusion:**

Our study suggests that LA-ESCC patients with lower pre-treatment HALP scores exhibit improved pathological response and reduced recurrence rate. As a comprehensive index of inflammatory nutritional status, pre-treatment HALP may be a reliable prognostic marker in ESCC patients undergoing neoadjuvant chemoimmunotherapy.

## Introduction

Esophageal cancer, a prominent cause of mortality among digestive tract malignancies, was the sixth leading cause of cancer-associated deaths worldwide in 2020, accounting for 544,000 fatalities (3.1% of all cancer deaths) (1). Esophageal cancer includes adenocarcinoma (AC) and squamous cell carcinoma (SCC) (2). Notably, esophageal squamous cell carcinoma (ESCC) comprises over 90% of cases in China (3, 4). Frequently diagnosed at a locally advanced stage, esophageal cancer poses significant treatment challenges (5). While surgical resection is the primary treatment for locally advanced cases, the 5-year survival rates remain suboptima (6, 7). Consequently, neoadjuvant therapy is recommended by the Chinese Society of Clinical Oncology (CSCO) and the National Comprehensive Cancer Network (NCCN) to enhance survival in patients with locally advanced ESCC (LA-ESCC) (8).

Immune checkpoint blockers (ICB) therapy has significantly progressed in treating metastatic or advanced ESCC (9, 10). The NCCN guidelines now endorse immunotherapy for treating advanced esophageal cancer (EC) (11). Immune checkpoint inhibitors (ICIS), which can induce systemic anti-tumor immunity by releasing new antigens from dead tumor cells and stimulating the initiation and expansion of new antigen-specific T cells in tumors before surgical resection, have advantages over adjuvants in preclinical studies (12). Recent studies indicate that neoadjuvant chemoimmunotherapy is a viable and safe option for LA-ESCC patients, enhancing major pathological response (MPR) and pathological complete response (pCR) with manageable adverse effects (13–15). However, research on predictive factors for pathological response in LA-ESCC patients undergoing neoadjuvant chemoimmunotherapy remains scant, and survival prognosis in this demographic is underexplored. Presently, there is an absence of reliable pre-treatment indicator to predict the pathological response and survival in these patients. Therefore, identifying cost-effective and practical pre-treatment indicators is imperative for prognostication in LA-ESCC patients before neoadjuvant chemoimmunotherapy.

Increasing evidence associates systemic inflammation and nutritional scores with cancer outcomes (16, 17). Inflammation-related indicators, such as the platelet-to-lymphocyte ratio (PLR), lymphocyte-to-monocyte ratio (LMR), and neutrophil-to-lymphocyte ratio (NLR), are increasingly utilized to forecast various cancer prognoses, including tumor survival or pathological complete response (pCR) post-neoadjuvant therapy (18, 19). Furthermore, comprehensive blood indicators like the systemic inflammation response index (SIRI), systemic immune-inflammation index (SII), prognostic nutritional index (PNI), and hemoglobin, albumin, lymphocyte, and platelet (HALP), are progressively applied in cancer prognosis evaluation (20–23). However, limited research has confirmed the impact of inflammation-related indicators on the prognosis of LA-ESCC patients receiving neoadjuvant chemoimmunotherapy.

Hence, this study aims to investigate the role of inflammation and nutritional status in predicting pathological response and disease-free survival (DFS) in LA-ESCC patients undergoing radical surgery following neoadjuvant chemoimmunotherapy. The objective is to explore more cost-effective and efficient pre-treatment indicators for prognosis prediction in LA-ESCC patients, ultimately enhancing the postoperative prognosis management of esophageal cancer.

## Methods

### Patient selection and data collection

The study was conducted following the Declaration of Helsinki and was approved by the Ethics Committee of Zhongshan Hospital Xiamen University. We conducted a systematic review of the clinical data of patients with locally advanced ESCC who underwent neoadjuvant chemoimmunotherapy. Before treatment, comprehensive diagnostic evaluations were performed, encompassing electronic gastroscopy, chest and abdominal CT scans, upper gastrointestinal angiography, neck color ultrasound, and PET-CT scans as needed. Inclusion criteria of this research were: patients receiving treatment at Zhongshan Hospital Xiamen University, Fujian Medical University Union Hospital, Affiliated Hospital of Putian University, Union Hospital as well as Thoracic Surgery from January 2019 to April 2023; age between 18 and 75 years old; resectable stage II-IVa ESCC as determined by the American Joint Committee on Cancer (AJCC) 8th edition TNM staging system; underwent radical esophagectomy; completeness of medical records and follow-up. Exclusion criteria encompassed a pathological diagnosis of adenocarcinoma or other types of esophageal carcinoma types; tumor location at the esophagogastric junction or gastric proximal; cancer complicated with malignant diseases of other organs or previous therapy (including radiotherapy, chemotherapy, and submucosal resection); autoimmune disease or hematologic disease; heart, lung, or liver dysfunction, or acute infection.

### Treatment and follow-up

Similar to our earlier research, the preoperative neoadjuvant chemoimmunotherapy treatment protocols were used (24). Patients received a minimum of two cycles of therapy, combining paclitaxel-based chemotherapy and PD-1 inhibitors. The PD-1 monoclonal antibodies used in this study included camrelizumab, pembrolizumab, sintilimab, tislelizuma, and toripalimab, administered at a dose of 200mg. Surgical resection followed 4-8 weeks post-treatment completion. Our department utilized the McKeown minimally invasive esophagectomy technique, including two-field lymphadenectomy and stomach-based digestive tract reconstruction. In cases of cervical lymph node involvement, a three-field lymphadenectomy was performed. Until now, the optimal adjuvant therapy following neoadjuvant chemoimmunotherapy and surgical intervention for patients with EC has yet to be clearly defined. The adjuvant treatment regimen varied among different institutions and involved the administration of adjuvant immunotherapy, radiotherapy, or chemotherapy. All patients were subject to regular follow-up assessments following the completion of their respective treatment modalities. Post-operation follow-up was performed every three months for the first three years, every six months for the subsequent three years, and the year after the fifth year. Follow-up procedures included routine blood examination, liver and kidney function, tumor markers and other laboratory tests, chest and abdominal CT scans, neck color ultrasound, upper gastrointestinal angiography, whole-body PET-CT, and bone scans. Magnetic resonance imaging was conducted and provided the needful. The AJCC/UICC 8th edition staging system and the modified Ryan protocol tumor regression grade (TRG) were used for evaluation (25). Major pathological response (MPR) was defined as the presence of less than 10% viable tumor cells in the resected tumor specimen, whereas pathological complete response (pCR) was defined as the absence of residual tumor cells in both the primary tumor and lymph nodes. Any recurrence, whether local, regional, or distant, was considered as such. Disease-free survival (DFS) was defined as the period from surgery until the first confirmed recurrence or death from any cause. The final follow-up period for this study concluded in June 2023.

### Definition of inflammation and nutritional scores

In this study, data acquisition was sourced from the medical record systems of the participating units. Laboratory tests included neutrophil count (NEUT), lymphocyte count (LY), monocyte count (MONO), hemoglobin (HB), albumin (ALB), and platelets (PLT). Additionally, platelet-to-lymphocyte ratio (PLR), lymphocyte-to-monocyte ratio (LMR), and neutrophil-to-lymphocyte ratio (NLR) were calculated. The hemoglobin, albumin, lymphocyte, and platelet (HALP) were calculated using the following formula: HALP = HB×ALB×LY/PLT. The systemic immune-inflammation index (SII) was calculated using the following formula: SII = PLT×NEUT/LY. The systemic inflammation response index (SIRI) was calculated using the following formula: SIRI = MONO×NEUT/LY. The prognostic nutritional index (PNI) was calculated using the following formula: PNI = ALB (g/L) + 5×LY (10^9^/L) (26, 27).

### Statistical analysis

Data analysis utilized SPSS 22.0 and R version 4.2.3. Normally distributed variables were presented as absolute numbers and percentages, mean, and standard deviation, while nonparametric variables were reported as median and interquartile range. Categorical variables were assessed using chi-square or Fisher’s exact test, while continuous variables were compared using t-test or Mann-Whitney U test. The survminer package in R determined optimal cut-off values for PLR, LMR, NLR, SII, PNI, HALP, and SIRI. To identify potential predictors of MPR, we employed the least absolute shrinkage and selection operator (LASSO) logistic regression model, and these variables were included in a multivariate logistic analysis. To prevent multicollinearity, a stepwise regression analysis was employed. DFS was estimated using the Kaplan-Meier method, with survival differences assessed by a stratified log-rank test. Cox proportional-hazards model was employed for univariate and multivariate analyses on DFS, considering P<0.05 as statistically significant.

## Results

### Patient characteristics

The study enrolled 190 patients diagnosed with LA-ESCC, comprising 154 males (81.05%) and 36 females (18.95%), with a median age of 61.4 years (IQR: 57.0–67.0 years). Tobacco use was reported by 56.32% of the patients, while 30.53% had a history of alcohol consumption. Additionally, hypertension was present in 19.47% of the patients, and diabetes in 5.26%. The majority of primary tumors were located in the middle (53.16%) or lower portion (38.94%) thoracic esophagus. At diagnosis, 72.63% of the patients were at clinical stage III or IV, with only 27.37% diagnosed at stage II. 68.42% of the patients received two cycles of neoadjuvant chemoimmunotherapy. Post-neoadjuvant therapy, pCR was achieved in 17.38% of patients, and MPR in 44.78%.

Inflammation and nutrition scores were categorized into low (L) and high (H) groups based on predetermined cutoff values(**Supplementary Table 1**). This categorization resulted in 30 patients (15.79%) in the L-PLR group,160 patients (84.21%) in the H-PLR group;43 patients (22.63%) in the L-NLR group, 147 patients (77.37%) in the H-NLR group; 165 patients (86.84%) in the L-LMR group, 25 patients (13.16%) in the H-LMR group; 157 patients (82.63%) in the L-HALP group, 33 patients (17.37%) in the H-HALP group; 33 patients (17.37%) in the L-SII group, 157 patients (82.63%) in the H-SII group; 154 patients (81.05%) in the L-SIRI group, 36 patients (18.95%) in the H-SIRI group; and 157 patients (82.63%) in the L-PNI group, 33 patients (17.37%) in the H-PNI group. The clinical and pathological characteristics of LA-ESCC patients are summarized in **Table 1**.

**Table 1:** Clinical characteristics in 190 esophageal squamous cell carcinoma patients.

Characteristics		Sum (n=190)
Sex, n (%)	Female	36 (18.95)
	Male	154 (81.05)
Age, n (%)	<65years	117 (61.58)
	≥65years	73 (38.42)
BMI, n (%)	<18.5kg/m2	24 (12.63)
	≥18.5kg/m2	166 (87.37)
ASA score, n (%)	I/II	168 (88.42)
	III	22 (11.58)
Smoking history, n (%)	No	83 (43.68)
	Yes	107 (56.32)
Drinking history, n (%)	No	132 (69.47)
	Yes	58 (30.53)
History of hypertension, n (%)	No	153 (80.53)
	Yes	37 (19.47)
Diabetes history, n (%)	No	180 (94.74)
	Yes	10 (5.26)
Clinical stage, n (%)	II	52 (27.37)
	III	116 (61.05)
	IV	22 (11.58)
Tumor location, n (%)	Upper	15 (7.89)
	Middle	101 (53.16)
	Lower	74 (38.95)
Immunotherapy drug, n (%)	Sintilimab	49 (25.79)
	Camrelizumab	68 (35.79)
	Tislelizumab	25 (13.16)
	Toripalimab	14 (7.37)
	Pembrolizumab	34 (17.90)
Neoadjuvant treatment cycles, n (%)	2 cycles	130 (68.42)
	>2 cycles	60 (31.58)
Time to surgery, n (%)	<6 weeks	97 (51.05)
	≥6 weeks	93 (48.95)
ypT, n (%)	1-2	113 (59.47)
	3-4	77 (40.53)
ypN, n (%)	N0	94 (49.47)
	N+	96 (50.53)
ypTNM stage, n (%)	I	67 (35.26)
	II/III/IV	123 (64.74)
pCR, n (%)	No	157 (82.63)
	Yes	33 (17.38)
MPR, n (%)	No	105 (55.26)
	Yes	85 (44.78)
HALP, n (%)	Low	157 (82.63)
	High	33 (17.37)
SII, n (%)	Low	33 (17.37)
	High	157 (82.63)
SIRI,n (%)	Low	154 (81.05)
	High	36 (18.95)
PNI, n (%)	Low	157 (82.63)
	High	33 (17.37)
LMR, n (%)	Low	165 (86.84)
	High	25 (13.16)
PLR, n (%)	Low	30 (15.79)
	High	160 (84.21)
NLR, n (%)	Low	43 (22.63)
	High	147 (77.37)
ALB, median [IQR]		41.10 [38.60,43.70]
NEUT, median [IQR]		4.34 [3.38,5.23]
LY, median [IQR]		1.69 [1.36,2.08]
MONO, median [IQR]		0.43 [0.33,0.54]
HB, median [IQR]		138.00 [128.00,145.00]
PLT, mean ( ± SD)		259.00 [211.00,295.00]
Pulmonary infection	No	137 (72.10)
	Yes	53 (27.90)
Anastomotic leakage	No	166 (87.37)
	Yes	24 (12.63)
Cardiac complication	No	159 (83.68)
	Yes	31 (16.32)
Pleural effusion	No	136 (71.58)
	Yes	54 (28.42)
Time of operation, min median [IQR]		330.00 [285.00,375.00]
Operative blood loss, ml median [IQR]		100.00 [100.00,200.00]

ASA score, the American Association of Anesthesiologists (ASA) classifies patients according to their physical condition and surgical risk. BMI, body mass index (kg/m^2^). pCR, pathological complete response. MPR, major pathological response. HALP,. SII, systemic immune-inflammation index. SIRI, systemic inflammation response index. PNI, prognostic nutritional index. LMR, Lymphocyte-to-monocyte ratio. PLR, platelet-to-lymphocyte. NLR, rationeutrophil-to-lymphocyte ratio. ALB, albumin. NEUT, neutrophil count, LY, lymphocyte count. MONO, monocyte count. HB, hemoglobin. PLT, platelets.

### Predictors for MPR using LASSO regression and logistic analysis

**Figure 1:**
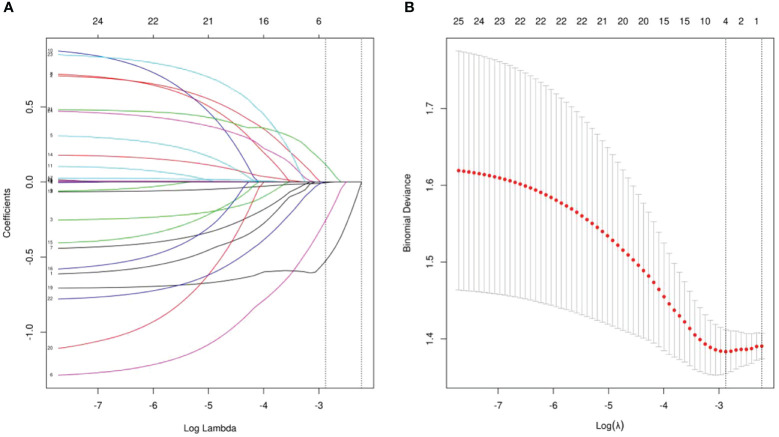
**(A)** LASSO coefficient profiles of the all indicators. **(B)** Ten-fold cross-validation for tuning parameter selection in the LASSO model.

The objective of this study was to identify prognostic indicators influencing MPR in patients with locally advanced ESCC undergoing neoadjuvant chemoimmunotherapy. We conducted a comprehensive analysis of the medical records of 190 patients, focusing on their general condition, coexisting diseases, specifics of the treatment received, and laboratory results. Employing LASSO regression, we identified several key factors, including HALP, SIRI, ASA status, and ALB(albumin), as significant predictors of MPR (**Figures 1A, B**). These factors were further examined through multivariate logistic regression analyses (**Table 2**). Our analysis highlighted HALP (OR = 0.335, 95% CI = 0.125,0.807, P = 0.02) as an independent predictor of MPR in this patient cohort. Additional statistical evaluations, including the Receiver Operating Characteristic curve (ROC), calibration curve, and Decision Curve Analysis (DCA), are detailed in the **Supplementary File**. The findings of our analyses are succinctly summarized in **Table 2**.

**Table 2:** Multivariable analysis of Predictors for MPR.

Variable	OR	95%CI	P-value
HALP	0.335	[0.125,0.807]	0.02
SIRI	1.674	[0.776,3.689]	0.192
ASA score	0.356	[0.111,0.976]	0.058
ALB	0.956	[0.883,1.028]	0.24

ASA score, the American Association of Anesthesiologists (ASA) classifies patients according to their physical condition and surgical risk. HALP, hemoglobin, albumin, lymphocyte, and platelet. SIRI, systemic inflammation response index. ALB, albumin. OR, odds ratio. 95%CI, 95% confidence interval.

### Patient characteristics grouped by HALP

This study focused on further validating the prognostic significance of the HALP in predicting DFS in ESCC patients treated with neoadjuvant chemoimmunotherapy. We utilized the Kaplan-Meier method and Cox regression analyses to assess the correlation between HALP and clinical outcomes. For this analysis, we included only those patients who had a follow-up period exceeding three months, resulting in a cohort of 181 patients. Within this group, 30 (16.57%) patients were classified under H-HALP, and 151 (83.43%) patients under L-HALP. Clinical characteristics grouped by HALP are presented in **Table 3**. Our findings indicated a significant association of L-HALP with MPR (P=0.007) and an association of H-HALP with high recurrence rates (P<0.001) and possibly with advanced ypTNM stages (P = 0.054). No significant differences were observed in other clinical information (P > 0.05). These results support the potential of HALP as a reliable prognostic marker for DFS in LA-ESCC patients undergoing neoadjuvant chemoimmunotherapy.

**Table 3:** Comparison of the clinical variables in esophageal squamous cell carcinoma grouped by HALP.

Variable		Sum (n=181)	L-HALP (n=151)	H-HALP (n=30)	P-value
Sex, n (%)	Female	35 (19.34)	30 (19.87)	5 (16.67)	0.685
	Male	146 (80.66)	121 (80.13)	25 (83.33)	
Age, n (%)	<65years	112 (61.88)	94 (62.25)	18 (60.00)	0.817
	≥65years	69 (38.12)	57 (37.75)	12 (40.00)	
BMI, n (%)	<18.5kg/m2	23 (12.71)	21 (13.91)	2 (6.67)	0.277
	≥18.5kg/m2	158 (87.29)	130 (86.09)	28 (93.33)	
ASA score, n (%)	I/II	163 (90.05)	137 (90.73)	26 (86.67)	0.497
	III	18 (9.95)	14 (9.27)	4 (13.33)	
Smoking history, n (%)	No	80 (44.20)	66 (43.71)	14 (46.67)	0.766
	Yes	101 (55.80)	85 (56.29)	16 (53.33)	
Drinking history, n (%)	No	126 (69.61)	105 (69.54)	21 (70.00)	0.96
	Yes	55 (30.39)	46 (30.46)	9 (30.00)	
History of hypertension, n (%)	No	149 (82.32)	125 (82.78)	24 (80.00)	0.715
	Yes	32 (17.68)	26 (17.22)	6 (20.00)	
Diabetes history, n (%)	No	173 (95.58)	143 (94.70)	30 (100.00)	nan
	Yes	8 (4.42)	8 (5.30)	0 (0.00)	
Clinical stage, n (%)	II	52 (28.73)	44 (29.14)	8 (26.67)	0.899
	III	108 (59.67)	89 (58.94)	19 (63.33)	
	IV	21 (11.60)	18 (11.92)	3 (10.00)	
Tumor location, n (%)	Upper	15 (8.29)	12 (7.95)	3 (10.00)	0.932
	Middle	93 (51.38)	78 (51.66)	15 (50.00)	
	Lower	73 (40.33)	61 (40.40)	12 (40.00)	
Immunotherapy drug, n (%)	Sintilimab	48 (26.52)	42 (27.82)	6 (20.00)	0.615
	Camrelizumab	65 (35.91)	52 (34.44)	13 (43.33)	
	Tislelizumab	23 (12.71)	21 (13.91)	2 (6.67)	
	Toripalimab	13 (7.18)	10 (6.62)	3 (10.00)	
	Pembrolizumab	32 (17.68)	26 (17.22)	6 (20.00)	
Neoadjuvant treatment cycles, n (%)	2 cycles	127 (70.17)	103 (68.21)	24 (80.00)	0.197
	>2 cycles	54 (29.83)	48 (31.78)	6 (20.00)	
Time to surgery, n (%)	<6 weeks	91 (50.28)	77 (50.99)	14 (46.67)	0.665
	≥6 weeks	90 (49.72)	74 (49.01)	16 (53.33)	
ypT, n (%)	1-2	108 (59.67)	94 (62.25)	14 (46.67)	0.112
	3-4	73 (40.33)	57 (37.75)	16 (53.33)	
ypN, n (%)	N0	91 (50.28)	79 (52.32)	12 (40.00)	0.218
	N+	90 (49.72)	72 (47.68)	18 (60.00)	
ypTNM stage, n (%)	I	64 (35.36)	58 (38.41)	6 (20.00)	0.054
	II/III/IV	117 (64.64)	93 (61.59)	24 (80.00)	
pCR, n (%)	No	149 (82.32)	123 (81.46)	26 (86.67)	0.494
	Yes	32 (17.68)	28 (18.54)	4 (13.33)	
MPR, n (%)	No	98 (54.14)	75 (49.67)	23 (76.67)	0.007
	Yes	83 (45.86)	76 (50.33)	7 (23.33)	
Recurrence, n (%)	No	152 (83.98)	133 (88.08)	19 (63.33)	<0.001
	Yes	29 (16.02)	18 (11.92)	11 (36.67)	
Pulmonary infection, n (%)	No	130 (71.82)	107 (70.86)	23 (76.67)	0.519
	Yes	51 (28.18)	44 (29.14)	7 (23.33)	
Anastomotic leakage, n (%)	No	157 (86.74)	130 (86.09)	27 (90.00)	0.564
	Yes	24 (13.26)	21 (13.91)	3 (10.00)	
Cardiac complication, n (%)	No	152 (83.98)	127 (84.11)	25 (83.33)	0.916
	Yes	29 (16.02)	24 (15.89)	5 (16.67)	
Pleural effusion, n (%)	No	128 (70.72)	104 (68.87)	24 (80.00)	0.221
	Yes	53 (29.28)	47 (31.13)	6 (20.00)	
Time of operation, min median [IQR]		327.00 [285.00,375.00]	320.00 [282.00,373.00]	334.00 [296.00,420.00]	0.132
Operative blood loss, ml median [IQR]		100.00 [100.00,200.00]	100.00 [100.00,200.00]	100.00 [100.00,200.00]	0.238

ASA score, the American Association of Anesthesiologists (ASA) classifies patients according to their physical condition and surgical risk. BMI, body mass index (kg/m^2^). pCR, pathological complete response. MPR, major pathological response. HALP, hemoglobin, albumin, lymphocyte, and platelet.

### Categorization of recurrence patterns in ESCC patients post-treatment

In this study, patients were stratified based on their initial post-treatment recurrence patterns into three distinct categories: local recurrence, distant metastasis, and concurrent local and distant metastasis. Among the 29 patients who experienced recurrence, 17 (58,62%) presented with local recurrence, which encompassed locoregional lymph node metastasis and anastomotic site recurrence. Additionally, 3 (10.34%) developed metastasis, while 9 (31.03%) patients exhibited both local recurrence and distant metastasis. Distant metastasis consisted of peritoneal metastasis and non-regional lymph node metastasis. Recurrence in two patients was confirmed through biopsy, whereas the remaining cases were identified via imaging examinations.

### HALP and disease-free survival of LA-ESCC

**Figure 2:**
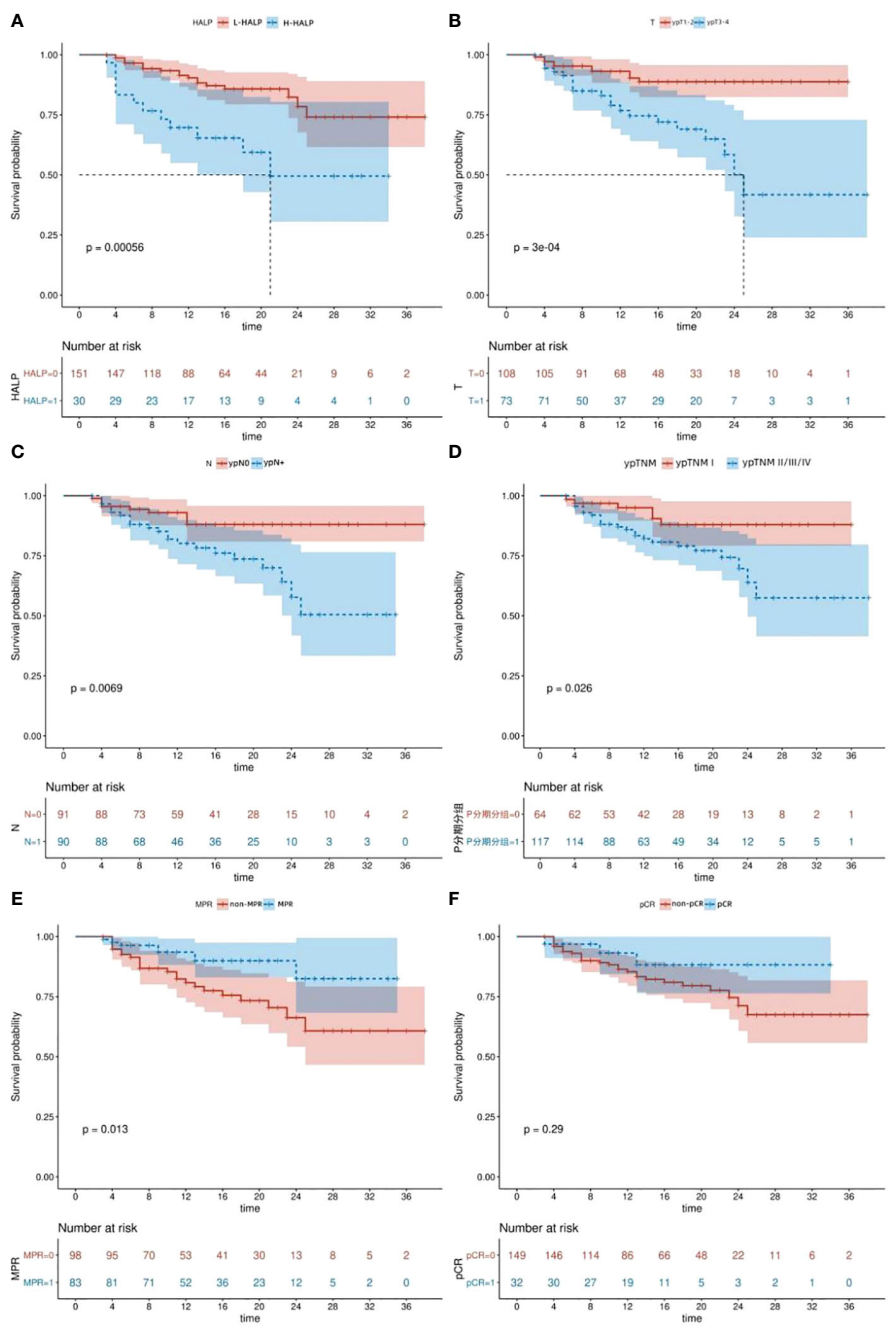
Kaplan-Meier survival curves. **(A)** DFS of patients with different HALP levels. **(B)** DFS of patients according to ypT stage. **(C)** DFS of patients according to ypN stage. **(D)** DFS of patients according to ypTNM stage. **(E)** DFS of patients according to MPR. **(F)** DFS of patients according to pCR. DFS, disease-free survival. pCR, pathological complete response. MPR, major pathological response. HALP, hemoglobin, albumin, lymphocyte, and platelet.

We aimed to explore prognostic factors influencing DFS in ESCC patients and further substantiate the predictive capacity of the HALP score. The median follow-up period was 16(range:3-38)months. Kaplan-Meier analysis revealed a significantly higher DFS in the L-HALP group compared to the H-HALP group (P=0.00056, **Figure 2A**). Additionally, we examined DFS variations across different postoperative pathological states. Our findings indicated that patients with MPR (P=0.013, **Figure 2E**) or ypTNM stage I (P=0.026, **Figure 2D**) exhibited extended DFS. Conversely, patients presenting with ypT3-4 (P=0.0003, **Figure 2B**) or ypN+ (P=0.0069, **Figure 2C**) had a poorer prognosis. Although not statistically significant, patients who achieved pCR showed a trend towards longer DFS (P=0.29, **Figure 2F**). Notably, the median DFS was reached only in the ypT3-4 and H-HALP subgroups, with durations of 25.0 and 21.0 months, respectively. In summary, our analysis suggests that HALP, ypT, ypN, ypTNM stage, and MPR are collectively indicative of survival outcomes in ESCC patients.

### Kaplan-Meier survival curves of L-HALP subgroup and H-HALP subgroup stratified by ypT stage

**Figure 3:**
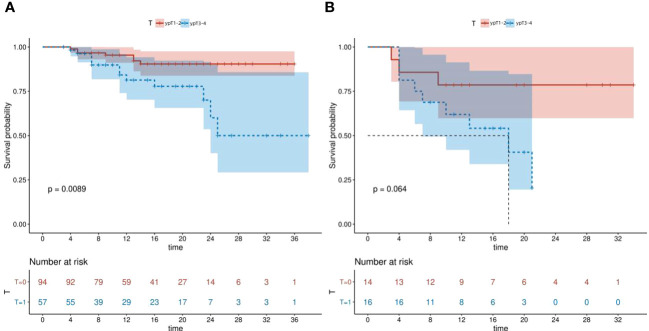
Kaplan-Meier survival curves of L-HALP subgroup and H-HALP subgroup stratified by ypT stage. **(A)** L-HALP subgroup. **(B)** H-HALP subgroup. HALP, hemoglobin, albumin, lymphocyte, and platelet.

The Kaplan-Meier survival curves were used to assess the DFS of the high and low HALP groups stratified by the ypT stage. Our findings demonstrated that patients within the L-HALP subgroup who were at ypTNM stage I stage exhibited significantly longer DFS (P=0.0089, **Figure 3A**). In contrast, while the H-HALP subgroup at ypTNM stage I also showed a trend towards extended DFS, this difference did not reach statistical significance (P=0.064, **Figure 3B**). Notably, only patients in the H-HALP subgroup with ypTNM II/III/IV stages achieved the median DFS, recorded at 18.0 months. Further investigations are warranted to confirm these observations.

### Univariate and multivariate Cox analysis of predictive factors for DFS

Univariate analysis (as shown in **Table 4**) was conducted to pinpoint predictive factors for DFS. The analysis indicated that H-HALP (HR: 3.3, 95% CI: 1.601,6.800, p=0.001), non-MPR (HR: 0.378, 95% CI: 0.169,0.846, p=0.018), ypT3-4 stage (HR: 3.643, 95% CI: 1.712,7.750, p=0.001),ypN+(HR: 2.773, 95% CI: 1.275,6.031, p=0.01),ypTNM stage II/III/IV (HR: 2.645, 95% CI: 1.082,6.467, p=0.033) and operative blood loss (HR: 1.003, 95% CI: 1.001,1.005, p=0.014) were significantly associated with a reduced DFS. Subsequently, a multivariate Cox regression analysis was performed to determine the independent predictive factors of DFS. The analysis underscored that patients in the H-HALP category exhibited a shorter DFS compared to those in the L-HALP (HR: 2.626, 95% CI: 1.175-5.865, p=0.019). Moreover, patients at ypT1-2 stages were found to have a better DFS than those at ypT3-4 stages(HR: 3.718, 95% CI: 1.145-12.069, p=0.029). These findings emphasize the independent prognostic significance of HALP and ypT stages in determining DFS among LA-ESCC patients.

**Table 4:** Univariate and multivariate analyses of DFS.

Variable		Univariable			Multivariable		
		HR	95%CI	P-value	HR	95%CI	P-value
Sex							
Female	35						
Male	146	1.404	[0.539,3.658]	0.488			
Age							
<65years	112						
≥65years	69	1.647	[0.810,3.351]	0.168			
BMI							
<18.5kg/m2	23						
≥18.5kg/m2	158	1.539	[0.366,6.464]	0.556			
Diabetes history							
No	173						
Yes	8	1.621	[0.385,6.835]	0.51			
History of hypertension							
No	149						
Yes	32	0.937	[0.359,2.448]	0.894			
Drinking history							
No	126						
Yes	55	1.241	[0.594,2.595]	0.566			
Smoking history							
No	80						
Yes	101	0.921	[0.455,1.865]	0.819			
ASA score							
I/II	163						
III	18	1.104	[0.335,3.634]	0.871			
Neoadjuvant treatment cycles							
2 cycles	127						
>2 cycles	54	1.188	[0.559,2.525]	0.654			
Time to surgery							
<6 weeks	91						
≥6 weeks	90	1.636	[0.800,3.346]	0.177			
Tumor location							
Upper	15						
Middle	93	2.479	[0.328,18.741]	0.379			
Lower	73	2.538	[0.331,19.453]	0.37			
Immunotherapy drug							
Sintilimab	48						
Camrelizumab	65	1.139	[0.475,2.733]	0.77			
Tislelizumab	23	0.617	[0.133,2.860]	0.538			
Toripalimab	13	0.791	[0.173,3.614]	0.763			
Pembrolizumab	32	0.966	[0.349,2.670]	0.946			
Clinical stage							
II	52						
III	108	2.301	[0.924,5.728]	0.073			
IV	21	1.634	[0.460,5.801]	0.447			
ypT							
1-2	108						
3-4	73	3.643	[1.712,7.750]	0.001	3.718	[1.145-12.069]	0.029
ypN							
N0	91						
N+	90	2.773	[1.275,6.031]	0.01	2.609	[0.916-7.431]	0.073
ypTNM stage							
I	64						
II/III/IV	117	2.645	[1.082,6.467]	0.033	0.438	[0.093-2.066]	0.297
pCR							
No	149						
Yes	32	0.53	[0.161,1.747]	0.297			
MPR							
No	98						
Yes	83	0.378	[0.169,0.846]	0.018	0.983	[0.336-2.876]	0.976
HALP							
Low	151						
High	30	3.3	[1.601,6.800]	0.001	2.626	[1.175-5.865]	0.019
Pulmonary infection							
No	130						
Yes	51	1.525	[0.730,3.187]	0.261			
Anastomotic leakage							
No	157						
Yes	24	1.126	[0.394,3.219]	0.825			
Cardiac complication							
No	152						
Yes	29	2.021	[0.903,4.520]	0.087			
Pleural effusion							
No	128						
Yes	53	1.04	[0.479,2.260]	0.921			
Time of operation	181	1.003	[0.999,1.007]	0.139			
Operative blood loss	181	1.003	[1.001,1.005]	0.014	1.001	[0.999-1.003]	0.464

ASA score, the American Association of Anesthesiologists (ASA) classifies patients according to their physical condition and surgical risk. BMI, body mass index (kg/m^2^). pCR, pathological complete response. MPR, major pathological response. HALP, hemoglobin, albumin, lymphocyte, and platelet. HR;hazard ratio. 95%CI, 95% confidence interval.

## Discussion

In recent years, the landscape of cancer treatment has increasingly pivoted towards immunotherapy, recognized as a breakthrough in therapeutic strategies. Compelling evidence from randomized phase III trials substantiates the efficacy of immunotherapy as a primary treatment modality either as a standalone approach or synergistically combined with chemotherapy, specifically for patients with advanced or metastatic esophageal squamous cell carcinoma (ESCC) (9, 10, 28). Building on this foundation, researchers have begun to explore the potential benefits of utilizing neoadjuvant chemotherapy combined with immunotherapy to treat locally advanced ESCC. Studies in this domain have revealed that adverse reactions associated with neoadjuvant immunotherapy are manageable, culminating in high incidences of R0 resection, commendable pCR rates, and limited postoperative complication (29, 30). However, the predictive factors influencing therapeutic outcomes remain elusive. With this in mind, the present investigation aims to identify vital prognostic factors that may impact MPR and DFS in patients with locally advanced ESCC treated with neoadjuvant chemoimmunotherapy.

Our investigation yielded that 17.38% of ESCC patients attained a pCR, while 44.78% achieved a MPR following neoadjuvant chemoimmunotherapy. In the current study, we initially explored the role of pre-treatment inflammatory nutritional scores on pathological response prediction in ESCC with neoadjuvant chemoimmunotherapy. Among the cohort with locally advanced ESCC undergoing neoadjuvant chemoimmunotherapy, we employed Lasso regression for an initial screening of potential predictive factors associated with MPR. This analysis pinpointed four pivotal predictors: the HALP, SIRI, ASA status, and serum albumin (ALB). Further, multifactor logistic regression analysis ratified HALP as an independent predictor of MPR.

Subsequently, we delved into the correlation between HALP and prognosis. To ascertain the prognostic significance of HALP and other variables for DFS, Kaplan-Meier curves and Cox regression analyses were utilized. Our results demonstrate that both HALP and ypT stage stand as robust prognostic markers for DFS in patients with LA-ESCC post-neoadjuvant chemoimmunotherapy. These insights furnish critical guidance for the implementation of chemoimmunotherapy in LA-ESCC treatment protocols. Markedly, this study is the first multicenter endeavor to evaluate the prognostic efficacy of the inflammatory-nutritional score in forecasting both pathological response and survival outcomes in LA-ESCC patients undergoing neoadjuvant chemoimmunotherapy.

Neutrophils are widely recognized as the primary source of vascular endothelial growth factor (VEGF), which can promote both tumor proliferation and neovascularization (31). Concurrently, there is increasing evidence of cancer progression being intertwined with the activation of circulating lymphocytes (32). Furthermore, a growing body of research underscores the complex roles of platelets in the advancement of cancer (33). In addition, composite inflammation and nutrition scores, derived from various hematological parameters, have been documented to possess prognostic significance across diverse cancer types, including ESCC (20, 21). The HALP score, which evaluates both immune and nutritional status, has emerged as a notable prognostic marker in various cancers, such as gastrointestinal and urogenital cancers (34, 35). Moreover, the Systemic Immune-Inflammation Index (SIRI), calculated using absolute neutrophil, monocyte, and platelet counts, has been identified as a prognostic element in patients with hepatocellular carcinoma and lung cancer (36, 37). Similarly, the lymphocyte-to-monocyte ratio (LMR) has been established as an independent predictor for achieving R0 resection in surgeries for hilar cholangiocarcinoma (38). Notably, while previous research has associated a low HALP score with poor survival, our study posits that a low HALP score may be a predictive factor for MPR and DFS in locally advanced ESCC patients undergoing neoadjuvant chemoimmunotherapy. This phenomenon warrants further exploration. Firstly, the precise role of platelets in tumor biology remains a subject of ongoing investigation. Some studies correlate an increase in pre-treatment platelet counts with enhanced neoadjuvant response (39). The team led by Tim F. Greten has found that platelets inhibit tumor growth and metastasis by releasing P2Y12-dependent CD40L (40). Others have found that an elevated baseline platelet count is an independent predictor of worse overall survival (41). Hence, the interaction between the bioactive factors released by platelets and the tumor immune environment in different situations remains to be studied. Platelet count is a critical component of HALP. Secondly, in this population of locally advanced ESCC patients receiving neoadjuvant chemoimmunotherapy, inflammatory factors might be activated during treatment, contributing to favorable responses (39). Such inflammatory states can precipitate the release of pro-inflammatory mediators from leukocytes, promoting lymphocyte apoptosis and platelet activation, while also impacting the metabolism of erythrocytes and albumin, potentially leading to reductions in the HALP score. Further studies are needed to confirm these findings and to elucidate the underlying mechanisms.

Additionally, our study suggests that in patients with ESCC undergoing neoadjuvant chemoimmunotherapy, ASA status may emerge as an independent predictor of MPR. The ASA score, a prevalent metric for evaluating preoperative physical status in surgical patients. not only reflects the patient’s overall health but has also been acknowledged as a reliable forecaster of postoperative complications and survival in various cancer types (42, 43). It is critical to highlight that despite employing stepwise regression analysis to mitigate multicollinearity, some degree of overfitting remain, attributed to the constrained sample size(OR: 0.356, 95% CI: 0.111,0.976, p=0.058). Consequently, future studies with larger cohorts are imperative to reinforce these findings more robustly.

Previous research indicates that ESCC patients achieving pathological remission post-neoadjuvant therapy substantially prolong survival (44, 45). Nevertheless, even in instances where patients attain pCR or MPR during surgery, the prospect of a complete cure remains uncertain. Moreover, it is observed that some patients may manifest a favorable prognosis without achieving pathological remission; hence, a multifaceted evaluation encompassing various factors is crucial for prognostic assessment. Therefore, our research also delves into the influence of disparate postoperative pathological outcomes and staging on DFS in LA-ESCC patients treated with neoadjuvant chemoimmunotherapy. Utilizing the Kaplan-Meier method for survival curves construction and the log-rank test for survival differences assessment, our findings reveal that patients with an MPR, ypT1-2, ypN0, or ypTNM I stage exhibit prolonged DFS (P<0.05). While patients with pCR appeared to have an extended DFS, the difference was insignificant (P>0.05). Moreover, multivariate Cox regression analysis identified ypT stage as an independent prognostic indicator for DFS in ESCC. Given the limitations of follow-up duration and sample size, subsequent research is warranted to explore the enduring impact of postoperative pathological outcomes and staging on survival.

Although our study identified HALP as a good prognostic factor for ESCC patients, several limitations exist. Primarily, the small sample size and the retrospective nature of this study constrain its broad applicability Consequently, the prognostic validity of the HALP score in ESCC patients undergoing neoadjuvant chemoimmunotherapy necessitates further corroboration in a more extensive cohort. Secondly, our investigation focused exclusively on the predictive value of inflammatory and nutritional scores, without delving into other potential prognostic indicators like tumor biomarkers. Thirdly, the optimal neoadjuvant treatment strategy for locally advanced ESCC remains a subject of ongoing debate. Therefore, our findings might not extend to other emerging treatment modalities, such as neoadjuvant radiochemotherapy combined with immunotherapy. Fourthly, the intricate mechanisms underpinning the relationship between inflammatory and nutritional scores and both pathological response and DFS require deeper exploration. Lastly, the relatively brief follow-up period of our study necessitates additional research to substantiate the long-term prognostic implications.

In summary, our research presents evidence that pre-treatment HALP scores can prognosticate MPR and DFS in locally advanced ESCC patients receiving neoadjuvant chemoimmunotherapy. Future studies are imperative to not only validate our findings but also to ascertain the clinical utility of HALP in guiding therapeutic decisions and enhancing patient outcomes.

## Data availability statement

The original contributions presented in the study are included in the article/**Supplementary Material**. Further inquiries can be directed to the corresponding authors.

## Ethics statement

Written informed consent was obtained from the individual(s) for the publication of any potentially identifiable images or data included in this article.

## Author contributions

JXX: Conceptualization, Data curation, Formal analysis, Funding acquisition, Investigation, Methodology, Project administration, Resources, Software, Supervision, Validation, Visualization, Writing – original draft, Writing – review & editing. YC: Conceptualization, Methodology, Visualization, Writing – original draft, Writing – review & editing. ZC: Methodology, Visualization, Writing – original draft, Writing – review & editing. JL: Conceptualization, Data curation, Methodology, Visualization, Writing – original draft, Writing – review & editing. XY: Writing – original draft, Writing – review & editing, Conceptualization, Data curation. SC: Conceptualization, Formal analysis, Funding acquisition, Investigation, Writing – original draft, Writing – review & editing. JBX: Resources, Software, Supervision, Validation, Writing – original draft, Writing – review & editing. MK: Conceptualization, Funding acquisition, Investigation, Methodology, Project administration, Resources, Software, Supervision, Validation, Visualization, Writing – original draft, Writing – review & editing. SK: Conceptualization, Data curation, Formal analysis, Funding acquisition, Investigation, Methodology, Project administration, Resources, Software, Supervision, Validation, Visualization, Writing – original draft, Writing – review & editing. ZH: Conceptualization, Data curation, Formal analysis, Funding acquisition, Investigation, Methodology, Project administration, Resources, Software, Supervision, Validation, Visualization, Writing – original draft, Writing – review & editing.

## References

[1] Tamandl D Paireder M Asari R Baltzer PA Schoppmann SF Ba-Ssalamah A. Markers of sarcopenia quantifed by computed tomography predict adverse long-term outcome in patients with resected oesophageal or gastro-oesophageal junction cancer. Eur Radiol (2016) 26:1359–67. doi: 10.1007/s00330-015-3963-1 26334504

[2] Cao W Chen HD Yu YW Li N Chen WQ. Changing profiles of cancer burden worldwide and in China: a secondary analysis of the global cancer statistics 2020. Chin Med J (2021) 134:783–91. doi: 10.1097/CM9.0000000000001474 PMC8104205 33734139

[3] Chen W Zheng R Baade PD Zhang S Zeng H Bray F. Cancer statistics in China, 2015. CA Cancer J Clin (2016) 66:115–32. doi: 10.3322/caac.21338 26808342

[4] Malhotra GK Yanala U Ravipati A Follet M Vijayakumar M Are C. Global trends in esophageal cancer. J Surg Oncol (2017) 115:564–79. doi: 10.1002/jso.24592 28320055

[5] Liu Y Li D-y. The progression of esophageal carcinoma immunotherapy. J Esophageal Dis (2022) 2:13–7.

[6] Guo X Mao T Gu Z Ji C Fang W. Clinical study on postoperative recurrence in patients with pN1 esophageal squamous cell carcinoma. Thorac Cancer (2015) 6:146–50. doi: 10.1111/1759-7714.12155 PMC4448496 26273351

[7] Ninomiya I Okamoto K Tsukada T Kinoshita J Oyama K Fushida S. Recurrence patterns and risk factors following thoracoscopic esophagectomy with radical lymph node dissection for thoracic esophageal squamous cell carcinoma. Mol Clin Oncol (2016) 4:278–84. doi: 10.3892/mco.2015.688 PMC4734145 26893875

[8] van Hagen P Hulshof MCCM van Lanschot JJB Steyerberg EW van Berge Henegouwen MI Wijnhoven BPL. Preoperative chemoradiotherapy for esophageal or junctional cancer. N Engl J Med (2012) 366:2074–84. doi: 10.1056/NEJMoa1112088 22646630

[9] Sun J-M Shen L Shah MA Enzinger P Adenis A Doi T. Pembrolizumab plus chemotherapy versus chemotherapy alone for first-line treatment of advanced oesophageal cancer (KEYNOTE-590): a randomised, placebo-controlled, phase 3 study. Lancet (2021) 398:759–71. doi: 10.1016/S0140-6736(21)01234-4 34454674

[10] Janjigian YY Shitara K Moehler M Garrido M Salman P Shen L. First-line nivolumab plus chemotherapy versus chemotherapy alone for advanced gastric, gastro-oesophageal junction, and oesophageal adenocarcinoma (CheckMate 649): a randomised, open-label, phase 3 trial. Lancet (2021) 398:27–40. doi: 10.1016/S0140-6736(21)00797-2 34102137 PMC8436782

[11] Esophageal and esophagogastric junction cancers. version 2 2021. Natl Compr Cancer Network 10.6004/jnccn.2011.0072 21900218

[12] Liu J Blake SJ Yong MCR Harjunpää H Ngiow SF Takeda K. Improved efficacy of neoadjuvant compared to adjuvant immunotherapy to eradicate metastatic disease. Cancer Discov (2016) 6:1382–99. doi: 10.1158/2159-8290.CD-16-0577 27663893

[13] Xu J Yan C Li Z Cao Y Duan H Ke S. Efficacy and safety of neoadjuvant chemoimmunotherapy in resectable esophageal squamous cell carcinoma: A meta-analysis. Ann Surg Oncol (2023) 30:1597–613. doi: 10.1245/s10434-022-12752-1 36380254

[14] Yan X Duan H Ni Y Zhou Y Wang X Qi H. Tislelizumab combined with chemotherapy as neoadjuvant therapy for surgically resectable esophageal cancer: A prospective, single-arm, phase II study (TD-NICE). Int J Surg (2022) 103:106680. doi: 10.1016/j.ijsu.2022.106680 35595021

[15] Wang Z Shao C Wang Y Duan H Pan M Zhao J. Efficacy and safety of neoadjuvant immunotherapy in surgically resectable esophageal cancer: A systematic review and meta-analysis. Int J Surg (2022) 104:106767. doi: 10.1016/j.ijsu.2022.106767 35840049

[16] Feng J Wang L Yang X Chen Q Cheng X. Pathologic complete response prediction to neoadjuvant immunotherapy combined with chemotherapy in resectable locally advanced esophageal squamous cell carcinoma: real-world evidence from integrative inflammatory and nutritional scores. J Inflamm Res (2022) 15:3783–96. doi: 10.2147/JIR.S367964 PMC9271687 35832830

[17] Coussens LM Werb Z. Inflammation and cancer. Nature (2002) 420:860–7. doi: 10.1038/nature01322 PMC2803035 12490959

[18] Eren T Karacin C Ucar G Ergun Y Yazici O İmamoglu Gİ. Correlation between peripheral blood inflammatory indicators and pathologic complete response to neoadjuvant chemotherapy in locally advanced breast cancer patients. Medicine (2020) 99:e20346. doi: 10.1097/MD.0000000000020346 32481414 PMC12245315

[19] Mandaliya H Jones M Oldmeadow C Nordman II. Prognostic biomarkers in stage IV non-small cell lung cancer (NSCLC): neutrophil to lymphocyte ratio (NLR), lymphocyte to monocyte ratio (LMR), platelet to lymphocyte ratio (PLR) and advanced lung cancer inflammation index (ALI). Trans Lung Cancer Res (2019) 8:886. doi: 10.21037/tlcr PMC6976360 32010567

[20] Chen J-H Zhai E-T Yuan Y-J Wu K-M Xu J-B Peng J-J. Systemic immune-inflammation index for predicting prognosis of colorectal cancer. World J Gastroenterol (2017) 23:6261. doi: 10.3748/wjg.v23.i34.6261 28974892 PMC5603492

[21] Okadome K Baba Y Yagi T Kiyozumi Y Ishimoto T Iwatsuki M. Prognostic nutritional index, tumor-infiltrating lymphocytes, and prognosis in patients with esophageal cancer. Ann Surg (2020) 271:693–700. doi: 10.1097/SLA.0000000000002985 30308614

[22] Wang J Jiang P Huang Y Tu Y Zhou Q Li N. Prognostic value of the cutoffs for HALP in endometrial cancer. Am J Clin Oncol (2023) 46:107–13. doi: 10.1097/COC.0000000000000977 PMC9946170 36700534

[23] Chao B Ju X Zhang L Xu X Zhao Y. A novel prognostic marker systemic inflammation response index (SIRI) for operable cervical cancer patients. Front Oncol (2020) 10:766. doi: 10.3389/fonc.2020.00766 32477958 PMC7237698

[24] Hong ZN Zhang Z Chen Z Weng K Peng K Lin J. Safety and feasibility of esophagectomy following combined neoadjuvant immunotherapy and chemotherapy for locally advanced esophageal cancer: a propensity score matching. Esophagus (2022) 19:224–32. doi: 10.1007/s10388-021-00899-x 34988773

[25] Ryan R Gibbons D Hyland JM Treanor D White A Mulcahy HE. Pathological response following long-course neoadjuvant chemoradiotherapy for locally advanced rectal cancer. Histopathology (2005) 47:141–6. doi: 10.1111/j.1365-2559.2005.02176.x 16045774

[26] Eraslan E Adas YG Yildiz F Gulesen AI Karacin C Arslan UY. Systemic immune-inflammation index (SII) predicts pathological complete response to neoadjuvant chemoradiotherapy in locally advanced rectal cancer. J Coll Physicians Surg Pak (2021) 30:399–404. doi: 10.29271/jcpsp 33866724

[27] Wu Y Li J. Change in maximal esophageal wall thickness provides prediction of survival and recurrence in patients with esophageal squamous cell carcinoma after neoadjuvant chemoradiotherapy and surgery. Cancer Manag Res (2021) 13:2433–45. doi: 10.2147/CMAR.S295646 PMC7979351 33758542

[28] Thuss-Patience P Stein A. Immunotherapy in squamous cell cancer of the esophagus. Curr Oncol (2022) 29:2461–71. doi: 10.3390/curroncol29040200 PMC9026413 35448174

[29] Shen D Chen Q Jie W Li J Tao K Jiang Y. The safety and effificacy of neoadjuvant PD-1 inhibitor with chemotherapy for locally advanced esophageal squamous cell carcinoma. J Gastrointest Oncol (2021) 12:1–10. doi: 10.21037/jgo 33708420 PMC7944149

[30] Shang X Zhao G Liang F Zhang C Zhang W Liu L. Safety and effectiveness of pembrolizumab combined with paclitaxel and cisplatin as neoadjuvant therapy followed by surgery for locally advanced resectable (stage III) esophageal squamous cell carcinoma: a study protocol for a prospective, single-arm, single-center, openlabel, phase-II trial (Keystone-001). Ann Transl Med (2022) 10:229. doi: 10.21037/atm 35280363 PMC8908169

[31] Kusumanto YH Dam WA Hospers GA Meijer C Mulder NH. Platelets and granulocytes, in particular the neutrophils, form important compartments for circulating vascular endothelial growth factor. Angiogenesis (2003) 6:283–7. doi: 10.1023/B:AGEN.0000029415.62384.ba 15166496

[32] Wang Y-Y Zhou N Liu H-S Gong X-L Zhu R Li X-Y. Circulating activated lymphocyte subsets as potential blood biomarkers of cancer progression. Cancer Med (2020) 9:5086–94. doi: 10.1002/cam4.3150 PMC7367640 32459060

[33] Ravindranathan D Master VA Bilen MA. Inflammatory markers in cancer immunotherapy. Biology (2021) 10:325. doi: 10.3390/biology10040325 33924623 PMC8069970

[34] Zhao Z Yin X-N Wang J Chen X Cai Z-L Zhang B. Prognostic significance of hemoglobin, albumin, lymphocyte, platelet in gastrointestinal stromal tumors: A propensity matched retrospective cohort study. World J Gastroenterol (2022) 28:3476–87. doi: 10.3748/wjg.v28.i27.3476 PMC9346454 36158264

[35] Peng D Zhang C-J Gong Y-Q Hao H Guan B Li X-S. Prognostic significance of HALP (hemoglobin, albumin, lymphocyte and platelet) in patients with bladder cancer after radical cystectomy. Sci Rep (2018) 8:1–9. doi: 10.1038/s41598-018-19146-y 29335609 PMC5768698

[36] Wang D Hu X Xiao L Long G Yao L Wang Z. Prognostic nutritional index and systemic immune-inflammation index predict the prognosis of patients with HCC. J Gastrointestinal Surg (2021) 25:421–7. doi: 10.1007/s11605-019-04492-7 PMC7904713 32026332

[37] Liu J Li S Zhang S Liu Y Ma L Zhu J. Systemic immune-inflammation index, neutrophil-to-lymphocyte ratio, platelet-to-lymphocyte ratio can predict clinical outcomes in patients with metastatic non-small-cell lung cancer treated with nivolumab. J Clin Lab Anal (2019) 33:e22964. doi: 10.1002/jcla.22964 31282096 PMC6805305

[38] Peng D Lu J Hu H Li B Ye X Cheng N. Lymphocyte to monocyte ratio predicts resectability and early recurrence of Bismuth-Corlette type IV hilar cholangiocarcinoma. J Gastrointestinal Surg (2020) 24:330–40. doi: 10.1007/s11605-018-04086-9 PMC7026310 30671792

[39] Ilhan-Mutlu A Starlinger P Perkmann T Schoppmann SF Preusser M Birner P. Plasma fibrinogen and blood platelet counts are associated with response to neoadjuvant therapy in esophageal cancer. Biomarkers Med (2015) 9:327–35. doi: 10.2217/bmm.14.111 25808437

[40] Ma C Fu Q Diggs LP McVey JC McCallen J Wabitsch S. Platelets control liver tumor growth through P2Y12-dependent CD40L release in NAFLD. Cancer Cell (2022) 40:986–998. e5. doi: 10.1016/j.ccell.2022.08.004 36055226 PMC9474605

[41] McLaren PJ Bronson NW Hart KD Vaccaro GM Gatter KM Thomas CR Jr. Neutrophil-to-lymphocyte and platelet-to-lymphocyte ratios can predict treatment response to neoadjuvant therapy in esophageal cancer. J Gastrointestinal Surg (2017) 21:607–13. doi: 10.1007/s11605-016-3351-4 28083838

[42] Park J-H Kim D-H Kim B-R Kim Y-W. The American Society of Anesthesiologists score influences on postoperative complications and total hospital charges after laparoscopic colorectal cancer surgery. Medicine (2018) 97(18):e0653. doi: 10.1097/MD.0000000000010653 29718883 PMC6393129

[43] Kang HW Seo SP Kim WT Kim YJ Yun SJ Lee SC. Impact of the ASA physical status score on adjuvant chemotherapy eligibility and survival of upper tract urothelial carcinoma patients: a multicenter study. J Korean Med Sci (2017) 32(2):335–342. doi: 10.3346/jkms.2017.32.2.335 PMC5220002 28049247

[44] Donahue JM Nichols FC Li Z Schomas DA Allen MS Cassivi SD. Complete pathologic response after neoadjuvant chemoradiotherapy for esophageal cancer is associated with enhanced survival. Ann Thorac Surg (2009) 87:392–9. doi: 10.1016/j.athoracsur.2008.11.001 PMC2930775 19161745

[45] Shen J Kong M Yang H Jin K Chen Y Fang W. Pathological complete response after neoadjuvant treatment determines survival in esophageal squamous cell carcinoma patients (NEOCRTEC5010). Ann Trans Med (2021) 9(20):1516. doi: 10.21037/atm PMC8576689 34790722

