# Supplementary material for: Prognostic value of inflammatory nutritional scores in locally advanced esophageal squamous cell carcinoma patients undergoing neoadjuvant chemoimmunotherapy: a multicenter study in China
Source: Front Oncol. 2024 Feb 15;14:1279733. doi: 10.3389/fonc.2024.1279733 (PMC10923400; doi:10.3389/fonc.2024.1279733)
Supplement: Supplementary file 1 [file DataSheet_1.docx]

Supplementary table 1 Pre-determined cut-off values of Inflammatory Nutritional Scores

|  | PLR | NLR | LMR | HALP | SII | SIRI | PNI |
| --- | --- | --- | --- | --- | --- | --- | --- |
| cut-off values | 96.28252788 | 1.735849057 | 6.548387097 | 59.01478599 | 362.4793388 | 1.975 | 54.900 |

HALP:hemoglobin, albumin, lymphocyte, and platelet. SII:systemic immune-inflammation index. SIRI:systemic inflammation response index. PNI:prognostic nutritional index.LMR:Lymphocyte-to-monocyte ratio.PLR:platelet-to-

lymphocyte. NLR: rationeutrophil-to-lymphocyte ratio.

Supplementary Figure 1


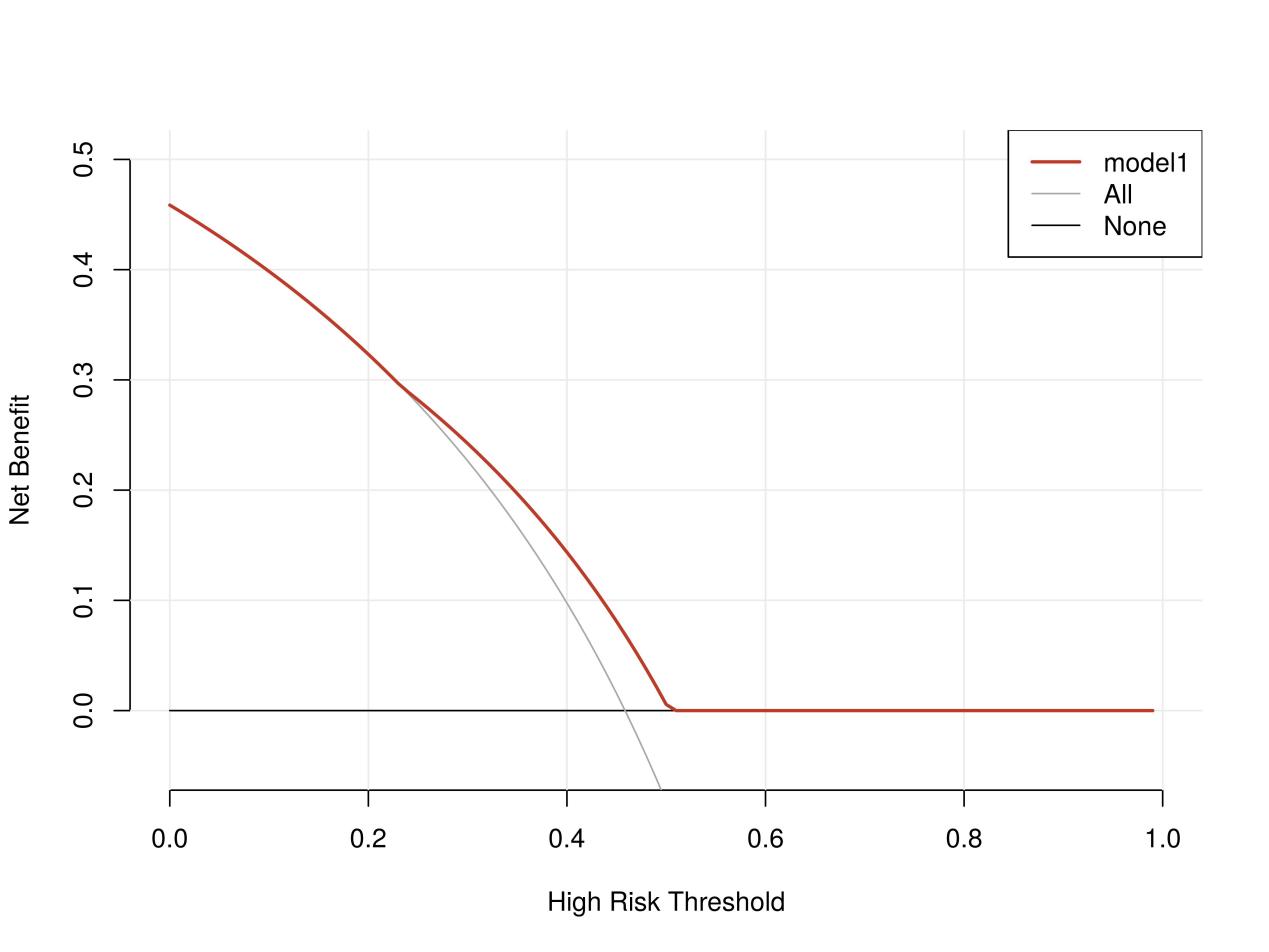


Decision curve analysis compares the net clinical benefits of three scenarios in predicting patient survival: a perfect prediction model (gray line), not screening patients at all (horizontal solid black line), and screening based on the HALP (red thick dash line).

Supplementary Figure 2


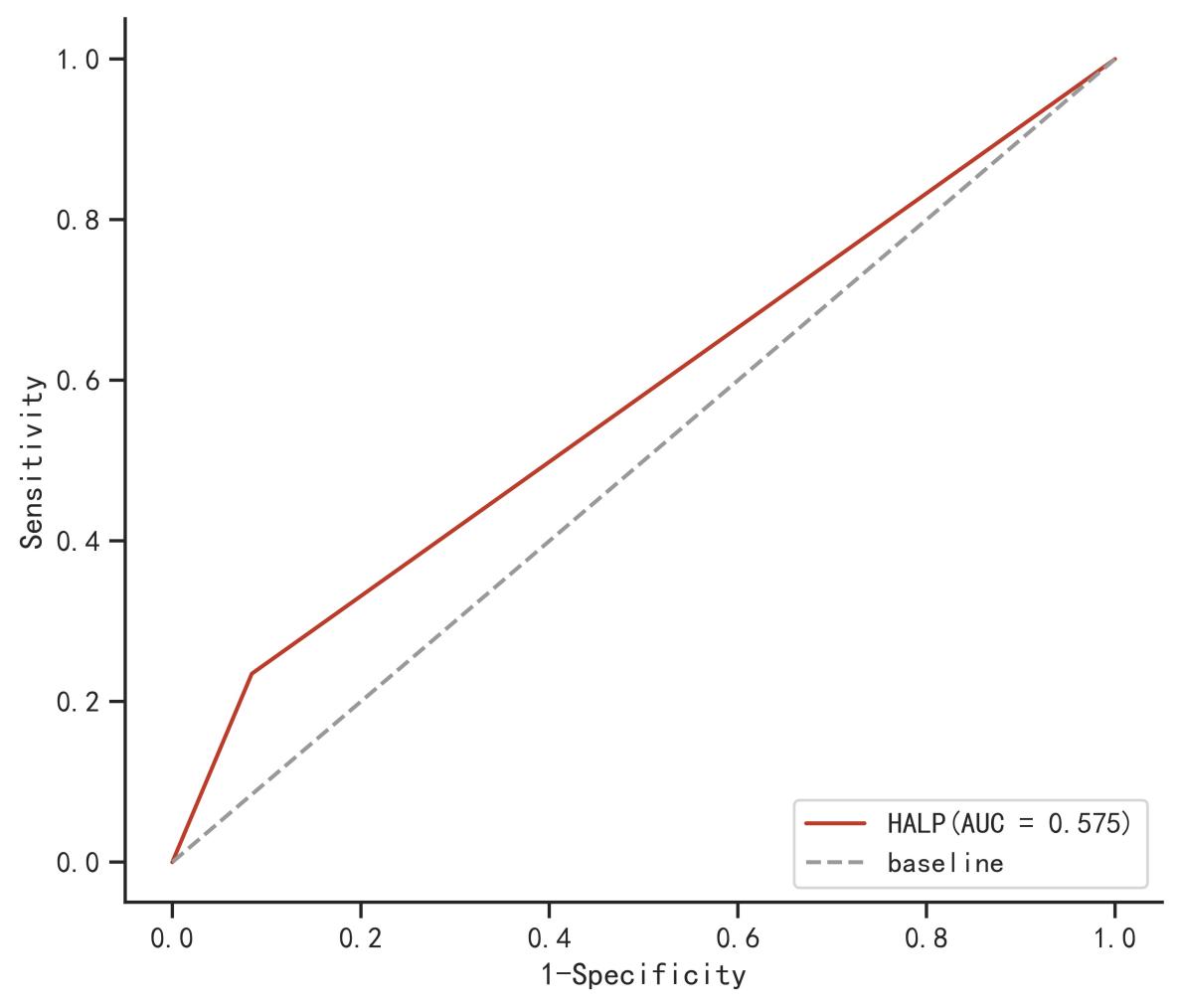


The receiver operating characteristic curve(ROC) and area under the ROC curve(AUC) of the model based on the HALP.

Supplementary Figure 3


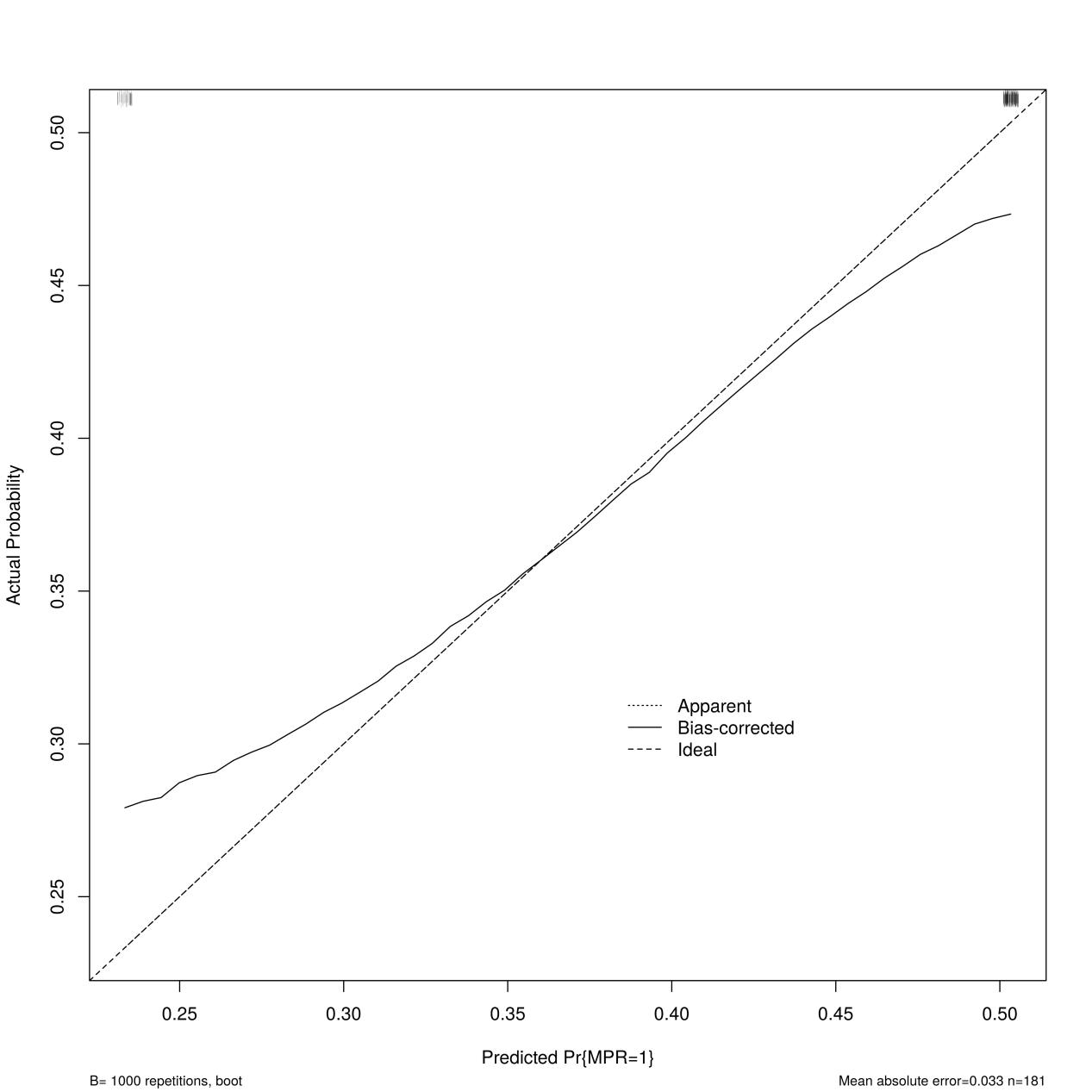


Calibration plot of the model based on the HALP.
